# Strait Gate: Special Issue on Advances in Silicon Chemistry

**DOI:** 10.3390/molecules22091497

**Published:** 2017-09-07

**Authors:** Mitsuo Kira

**Affiliations:** Key Laboratory of Organosilicon Chemistry and Material Technology of Ministry of Education, Hangzhou Normal University, Hangzhou 311121, Zhejiang, China; mkira@m.tohoku.ac.jp

Manufacturing high-purity element silicon and organic polysilicones are two major silicon industries, supporting the basis of the modern electronic industry and our daily lives. Nowadays, the use of element silicon and silicon compounds is spreading rapidly in many areas, including electronics, automobiles, textiles, healthcare, construction, and household products.

In parallel with the development of the silicon and silicone industries, scientific research in silicon chemistry has been growing rapidly in recent decades. Knowledge on a number of silicon reactive species, radicals, ions, divalent species, etc., has been accumulated and their properties have been compared with those of the corresponding carbon reactive intermediates that have been well documented on the basis of the systematic principles of physical organic chemistry. In 1981, R. West et al. [1] and A. G. Brook [2] synthesized isolable disilene and silaethene with sterically protecting substituents, respectively, as the first Si=Si and Si=C doubly bonded compounds. Before that, the dominant view maintained that these unsaturated compounds of silicon and other heavier main-group elements should be unstable and unisolable, according to the so-called classical double-bond rule [3,4]. Using a guiding principle that states that the sterically demanding substituents protect fragile bonds and electronic structures, a number of hitherto unknown or labile silicon compounds have been synthesized and characterized by means of various spectroscopies and X-ray crystallography. Among them are silylenes, trivalent silyl radicals, cation and anion structured disilenes (cyclotrisilenes, cyclotetrasilenes, spiropentasiladiene), Si=X doubly bonded compounds (X = S, Se, Te, N, P, Bi, etc.), 1,3-tetrasiladienes, trisilaallenes, disilynes, and so on. The coordination of *N*-hetrocyclic carbenes (NHCs) and other Lewis bases has been introduced as another lodestar to stabilize the fragile species more recently. According to the guide, the synthesis and isolation of many unusual Lewis-base stabilized silylenes have been achieved, such as L:→:SiCl_2_ (L: = an NHC), bissilylene (L:→:Si(Cl)–(Cl)Si:←:L), Si(0) disilene (L:→:Si=Si:←:L), base-stabilized Si=O compounds, and base-stabilized Si(0) compounds like L:→Si(0)←:L, etc. These discoveries have revealed not only that silicon constructs almost all types of compounds that carbon does, but also that silicon combines with other elements to form many types of structures otherwise unrealizable in carbon chemistry, in addition to the classically-found penta- and hexa-coordinate silicon compounds. No NHC-coordinate carbenes or Lewis-base-stabilized C(0) compounds have been known until now. Moreover, remarkable differences in the structural characteristics between carbon compounds and their silicon congeners have been revealed. For example, geometries around the Si=Si and Si≡Si bonds of disilene and disilylne are trans-bent in contrast to those around the C=C double and C≡C triple bonds. The geometry of a trisilaallene is not linear but bent. A cyclotetrasiladiene has been found to be rhombic by the Jahn-Teller distortion, while cyclobutadienes are well-known to form a rectangular ring by the distortion.

The development of silicon industries and the acquisition of the basic knowledge of the silicon chemistry represent two sides of the same coin. It is well known that in the early history of the silicon chemistry, F. S. Kipping’s classical studies [5] stimulated young researchers in companies to study polysilicones as an electric insulator and glass coating material, and contributed to the development polysilicone industry [6], which in turn instigated the progress of modern basic silicon chemistry. Many precursors necessary to this basic research have been supplied through the industry. Important contributions to the development of the basic silicon chemistry have been achieved by the researchers working in the industry, especially in its early stages. In addition to these, through the well-known story of Rochow-Müller’s discovery of the direct synthesis of chlorosilanes [6], not only corporate researchers but the basic silicon chemists have encouragingly learned how a truly-creative study is invented.

Under the current situation in the silicon chemistry, we are faced with two major questions: (1) Will new silicon industries rise in the future on the basis of the fruits of modern silicon chemistry? (2) What does the research on basic silicon chemistry aim for? The first question reminds us of the Bakerian lecture of Kipping in 1936 [7], who concluded the lecture with a somewhat pessimistic view of the future of the silicon chemistry; “—the prospect of any immediate and important advance in this section of organic chemistry does not seem to be very hopeful.” However, at the same period, in spite of his view, the abovementioned young corporate researchers caught a whiff of the promising future of the silicon chemistry and eventually launched the polysilicone industry. This story suggests that the new silicon industries will be created unexpectedly or accidentally by researchers with acute perceptiveness to the chemistry and the industry.

The second question will be worth considering more seriously. As mentioned above, various silicon compounds have been created and their unique bonding and structure have been analyzed by modern spectroscopies and sophisticated theoretical calculations. The research in this direction will continue being active and important for the future. However, only the accumulation of knowledge of the individual compounds will be insufficient to develop basic silicon chemistry as a branch of natural science. Why are disilenes trans-bent; while alkenes are planar? How should Si-carbene carbon bonds in the NHC-coordinate silylenes, disilenes, etc. be described, if not as a covalent bond? Why are the Si(0) compounds formed? The bonding and structural features have often been reproduced by the molecular orbital calculations, but the results have only secured that the features are able to be discussed in the framework of the molecular orbital theory. Qualitative explanation has been given sometimes individually, but not systematically. A dominating basic concept in the chemistry of heavy main-group elements, “hybridization defect” or “inert s pair effect” [8,9], has often been invoked, but in the indirect manner. It is desired that qualitative but highly systematic theories be brought forth, such as those established in physical organic chemistry. Enter by the narrow gate; this gate may lead to the general natural laws describing structural features of silicon and other heavy main-group elements.

A compilation of 12 full articles in the special issue titled “Advances in Silicon Chemistry” features currently developing areas in basic, applied, and theoretical silicon chemistry. Among them are studies of base-coordinate phosphasilenes [10], base-coordinate silyl cations [11], a new germylene [12], and σ-delocalization in structured oligosilanes [13] that encourage us to think seriously about the outlook of bonding and structure of heavy main-group elements. Hopefully, the readers will enjoy finding excellent articles in this special issue.
